# The effect of global price movements on the energy sector commodity on bitcoin price movement during the COVID-19 pandemic

**DOI:** 10.1016/j.heliyon.2022.e10820

**Published:** 2022-09-29

**Authors:** Meiryani Meiryani, Caineth Delvin Tandyopranoto, Jason Emanuel, A.S.L. Lindawati, Mochammad Fahlevi, Mohammed Aljuaid, Fakhrul Hasan

**Affiliations:** a Accounting Department, School of Accounting, Bina Nusantara University, Jakarta 11480, Indonesia; b Finance Program, Accounting Department, School of Accounting, Bina Nusantara University, Jakarta 11480, Indonesia; c Finance Program, Accounting Department, Faculty of Economics and Communication, Bina Nusantara University, Jakarta 11480, Indonesia; d Management Department, BINUS Online Learning, Bina Nusantara University, Jakarta 11480, Indonesia; e Department of Health Administration, College of Business Administration, King Saud University, Riyadh, Saudi Arabia; f Liverpool Hope University Business School, Liverpool Hope University, Hope Park, L16 9JD, Liverpool, UK

**Keywords:** Bitcoin, Crude oil, Natural gas, COVID-19, Energy

## Abstract

This study aims to determine the effect of global price movements for energy sector commodities, especially Crude Oil and Natural Gas Prices, on cryptocurrency price movements. This study focuses more on the Bitcoin cryptocurrency. This study uses quantitative methods, and the data collection used is secondary data with weekly data and the period from January 1, 2020–July 31, 2021. The number of observations used in this study amounted to 79 observations. Secondary data sources are obtained through the website finance.yahoo.com. The data processing technique will be carried out using Stata and SPSS software, the Multiple Linear Regression method, and the Classical Assumption Test. The results of this study show that global prices for energy sector commodities, especially Crude Oil, Natural Gas, have a positive effect on Bitcoin price movements. These results indicate a link between energy and Bitcoin caused by Bitcoin miners who are mining Bitcoin using energy so that when the price of Bitcoin rises, the price of energy will also increase.

## 1 Introduction

Virtual currency is a relatively new phenomenon in global finance. Therefore, identity, structure, and function are constantly improving; however, this medium is increasingly recognized as a better medium for global finance with great potential (Abboushi, 2017). Bitcoin was developed to avoid third parties such as banks, cards, and governments and to avoid delays and costs such as transferring money (Olvera-Juarez and Huerta-Manzanilla, 2019).

Initially, Bitcoin was planned to be able to make payments on the internet without limits and central authority fees. This allows Bitcoin to behave as an asset transfer analogy, i.e., retaining value on its own. Furthermore, at the same time, Bitcoin is defined as a money economy; that is, it is a medium of exchange, a unit of account, and a store of value (Nian and Chuen, 2015). Bitcoin transactions increased from 50 units in January 2009 to over 17 million, with an increase in transaction volume of $50 billion for all cryptocurrencies in December 2017. More than 100,000 companies now accept transactions in Bitcoin, and nearly 10 million people worldwide hold large sums of money in the form of Bitcoin as a financial asset (Hileman and Rauchs, 2017).

Bitcoin has been declared a commodity under the Commodity Exchange Act (CEA) since 2015. The treatment of Bitcoin as a commodity indicates that it is also sensitive to the rest of the commodities on the market and other macroeconomic indicators (Jalal et al., 2020). Bouri et al. (2020) also showed that Bitcoin is a strong hedge against energy commodities. The energy commodity in the form of Crude Oil influenced Bitcoin during the COVID-19 period (Okorie and Lin, 2020).

In addition, the interest of researchers in exploring the relationship between the cryptocurrency currency market and the commodity market is increasing (Ji et al., 2021). Goodell and Goutte (2020) suggest exploring economic research related to the COVID-19 period. Previous studies have been limited in discussing the relationship between cryptocurrency currencies and commodity markets during the COVID-19 period. Investors are more encouraged to review the long-term outlook for Bitcoin during the pandemic. Therefore, this study examines the relationship between commodities and Bitcoin during the pandemic.

Several phenomena affect Bitcoin as a cryptocurrency and commodity asset. When amid the rise of virtual currencies, the US Commodity Futures Trading Commission (CFTC) declared cryptocurrencies as commodities, thus implying that they were not currencies or investment vehicles but cryptocurrency commodities that could be traded in the natural or virtual world. Commodities and cryptocurrencies work similarly, as costs are associated with their creation and use. This has given rise to some arguments that cryptocurrencies represent the future of global commodity trading, and this may include oil as it is the most prominently traded commodity. Since there is an inverse correlation between the dollar and the price of oil, once the dollar weakens or falls, Oil prices move in the opposite direction. However, the correlation between cryptocurrencies and the oil market is not fully realized, as many emerging market countries influence oil. The author, an energy and oil marketing adviser, argues that cryptocurrencies will be very doubtful to be used as oil hedges and are very risky as a place to invest (Faeq, 2021).

Then the next case was after South Korea announced a plan to ban cryptocurrency money trading; the price of Bitcoin fell below the US $ 13,000 on January 11, 2018. Lukman Otunuga, a Research Analyst at FXTM, said that Bitcoin looks to be under pressure on the daily chart. This continued decline could lead to continuous depreciation towards US$12,000, then US$10,000. On the other hand, world oil prices began to soar to their highest levels, although there were fears of a rally when they began to weaken. "The price of WTI Crude Oil entered a level that has not been touched since December 2014 at $64 in the trading session,” said Lukman. Sentiment improved as crude oil inventories in the US fell, indicating a significant supply disruption; geopolitical risks and optimism that OPEC's production cuts can balance the market are some factors causing the rise in oil prices. Oil prices may continue to strengthen due to the current market optimism, but keep in mind that increased US Crude Oil production could expose oil to downside risks (Bisnis.tempo.co, 2018).

Then another phenomenon is the increase in the price of Bitcoin and other cryptocurrencies, causing concerns about the intensive use of energy required to obtain it. For the US company, EZ Blockchain, the energy source from Natural Gas, is the energy source for mining Bitcoin and other cryptocurrencies. According to Sergii Gerasymovych, CEO of EZ Blockchain, Natural Gas jets are the perfect power source, "I think the market is huge,” said Gerasymovych. His company has six data centers with Natural Gas sources in Utah, New Mexico, the USA, and Canada. However, adverse reactions have formed against the energy use of digital assets due to the dependence of these assets on carbon-generating resources that contribute to climate change. Some consider using wasted natural gas as a solution to this problem. However, according to Tony Scott, managing director of analytics at oil and gas research firm BTU Analytics, Natural Gas's ability to reduce emissions remains to be seen. "In the grand scheme of things and relative to other loads, yes, it is small,” said Scoot. "They create economic value (but) they do not necessarily change the emissions profile significantly.” (Koesanto, 2021).

The purpose of this research is to find out and understand more deeply the relationship between bitcoin and commodities, which in this study are crude oil and natural gas. Although it is a metaphor, bitcoin mining activity often equates bitcoin with other commodities, namely crude oil, where crude oil is mined to get it (Ozturk, 2020). These similarities can be investigated by analyzing the interrelationships between these assets, as it is known that Bitcoin market conditions are also complex and difficult to predict. Therefore, this research is motivated by the uncertain, unstable, complex Bitcoin market conditions and the elements of bitcoin which are similar to commodities, namely how to get them, by trying to analyze the relationship between the Crude Oil and Natural Gas commodity market and Bitcoin. We can develop the relationship between Bitcoin, Natural Oil, and Natural Gas from the methodology. For details, multiple linear regression and classical assumption tests are used to see if the data is correct and feasible to process and to see a deeper relationship between Bitcoin, Crude Oil, and Natural Gas. This research contributes to the literature on the cryptocurrency market, particularly bitcoin, in several aspects. First, our study offers a new and unprecedented research period, although several studies are also analyzing bitcoin and crude oil (Kaabia et al., 2020). However, no studies use the period during the Covid-19 pandemic. Second, our method differs from several previous studies, i.e., Ozturk (2020) and Kaabia et al. (2020). Multiple linear regression is used to determine the effect of the independent variable on the dependent variable and whether the two variables choose a positive or negative relationship. In this case, this research has profound implications in investment management in knowing the effect of crude oil and natural gas on bitcoin during the Covid-19 pandemic.

The results in this study indicate that during the Covid-19 pandemic, the commodity sector, which in this study is natural gas and crude oil, has an influence on bitcoin. During the Covid-19 period, the price of natural gas and the price of crude oil had a positive influence on the price of bitcoin. So, when the price of natural gas and crude oil rises, bitcoin prices rise and vice versa.

This research was prepared through a literature review, a research methodology, data analysis, and findings, followed by a discussion. In the end, conclusions are presented along with suggestions for readers and further researchers.

## 2 Literature review (and hypothesis development)

Cryptocurrency is a computer currency whose implementation is based on cryptography principles, which authorize transactions to produce new currencies. Cryptocurrencies usually use a proof-of-work scheme to ensure seller protection from fraud by recording all transactions in a public ledger. Cryptocurrencies, unlike Dollars or Euros, can have inflationary phenomena; they have no fundamental value. Most cryptocurrencies are designed to have an upper limit on the total amount of currency in circulation and gradually introduce new money. Bitcoin is one of the most prominent in terms of its tremendous market cap and price development (Letra, 2016). Unlike other digital currencies, which can be issued centrally, circulated within a community, or tied to fiat currencies or the organization that issues them, cryptocurrencies have very different characteristics. The technology called Blockchain used by cryptocurrencies is an open ledger that records all transactions and prevents double spending, and no third party is involved. Decentralization enables Blockchain technology to increase capacity, better security, and faster settlement (Lee et al., 2018).

Bitcoin is one of the pioneers in the world of cryptocurrency, which is included in the peer-to-peer payment network. Bitcoin has evolved to continuously meet market needs and foster a thriving cryptocurrency ecosystem (Hass, 2018). Not only exists as a currency, but Bitcoin is also now an investment choice despite the lack of understanding of Bitcoin (Urquhart, 2016). Bitcoin's ultimate goal, according to its proponents, is to serve as an alternative to existing payment systems and enable cross-border transactions and currency denominations without the intervention of sovereign entities or central banks and without alleged exploitation by traditional financial intermediaries such as banks (Lo and Wang, 2014). Bitcoin has no intrinsic value like gold because it cannot be used to make physical objects such as jewelry that have value. Nevertheless, value continues to exist because of trust and acceptance (DeVries, 2016).

Energy is produced by various types of natural resources, one of which is oil and gas (Goodstein, 2004). Crude oil is a hydrocarbon compound composed of the main elements, namely carbon, hydrogen, oxygen, nitrogen, and sulfur. Petroleum is formed from decomposing organic compounds from animals, plants, and microorganisms that died millions of years ago. The decomposition process takes place chemically and physically, and this process occurs at high temperatures and pressures (Ardiatma and Sasmita, 2019).

Crude oil can affect the conditions of economic growth. Crude oil is one of the most traded commodities, its price is very volatile, and it can have an impact on improving the stability of the global financial system (Luo and Ji, 2018).

Natural Gas is a mixture of gases that is rich in hydrocarbons. All these gases, such as methane, nitrogen, and carbon dioxide, are naturally found in the atmosphere. Natural Gas Reserves are deep in the earth near layers of other solid & liquid hydrocarbons such as coal and Crude Oil (TheEconomicTimes, 2021). Natural gas is a non-renewable resource made from fossils of living things trapped in deep rocks with high pressure and temperature. This natural gas cannot be found in all layers of the earth because it requires good porosity, and the layer must be perfectly insulated.

Natural Gas is considered an environmentally friendly clean fuel, offering critical environmental benefits compared to other fossil fuels. The superior environmental quality of coal or Crude Oil is that sulfur dioxide emissions are negligible and nitrous oxide and carbon dioxide emission levels are lower. This helps reduce the problem of acid rain, the ozone layer, or greenhouse gases. Natural Gas is also a very safe energy source when transported, stored, and used (Mokhatab and Poe, 2012).

Many previous studies have examined the relationship between crude oil and bitcoin. For example, Kaabia et al. (2020) used the Bayesian Shinkage VAR model and found a significant positive relationship between Bitcoin and all Crude Oil Prices. Evidence that Bitcoin affects Crude Oil Prices has been found. These results confirm that Crude Oil Prices are sensitive to price variations of Bitcoin, and therefore the entire energy industry will be affected by the Bitcoin Price crisis. Ozturk (2020) also researched the relationship between Bitcoin, Crude Oil, and Gold and found that the value of the relationship between these three variables measured by volatility could reach almost 70%. Even after that date, for 5–6 months, the connection between the assets increases and affects the connectedness between assets. Then when measured by return, the level of correlation is not as high as volatility, which is only about 5%–35%.

Okorie & Lin (2020) researched using the VAR MGARCH GJR–BEKK method and the Wald test to measure the volatility relationship between Crude Oil and Bitcoin. It was found that there is a two-way relationship and unidirectional volatility from the Crude Oil market to the Cryptocurrency market. They also found evidence for hedging Cryptocurrencies using Crude Oil, the potential for hedging from Crude Oil with Cryptocurrencies can be long-lived; therefore, Crude Oil in portfolio investment is the right strategy to diversify with Cryptocurrencies. Jin et al. (2019) researched which is more informative in determining price fluctuations using bitcoin, gold, and crude oil. They found the spillovers from the gold and crude oil markets to the Bitcoin market much stronger than the other spillovers. The dynamic correlation between Bitcoin and the crude oil market was also negative over the entire sample period. Selmi et al. (2018) examined the potential of hedging Bitcoin against Crude Oil price movements; the results found that Bitcoin can be used as a hedging instrument. However, examining the breakdown of Crude Oil price movements can be essential to understanding the nature of these price movements. So it is crucial to understand the nature of the movement by which bitcoin can be an effective hedge. Gajardo et al. (2018) also examine the relationship between bitcoin and commodities, especially crude oil, and various other financial markets, such as currency rates and the Dow Jones Industrial Average. During the period of the research conducted, i.e., 13/09/2005–25/08/17, it was found that Bitcoin exhibits a very different relationship with commodities and stock market indices and that they think these results should be taken into account when investing. Das et al. (2020) examined Bitcoin's hedging and safe-haven instruments against crude oil volatility and structural shocks using the GARCH dummy variable and quantile regression models. This study states that Bitcoin is not a superior asset to others to hedge oil-related uncertainties.

Ciaian et al. (2016) stated that the price of Crude Oil is considered a determinant of Bitcoin volatility. Vassiliadis (2017) also proves that Bitcoin has a cross-correlation with the price of Crude Oil and Gold. Van Wijk (2013) conducted a study examining the value of bitcoin using financial data, one of which was oil price. The oil prices used in this study are the Brent oil price, the West Texas Intermediate oil price, and the UBS Bloomberg Constant Maturity Commodity Index of Oil. The results of this study state that the price of WTI oil significantly influences the value of Bitcoin in the long term. Su et al. (2020) researched the causality or influence that the Bitcoin market has on the oil market on each other. The analysis in the study reveals that the influence emanating from the oil price and transmitted to the bitcoin price can be positive or negative. The positive impact shows that Bitcoin can be seen as an asset that helps avoid the risk of high oil prices, indicating that Bitcoin and oil are in a similar state. However, on the other hand, the negative effect of oil prices on bitcoin prices can be explained by the bursting of the Bitcoin bubble, which has weakened the hedging ability. Based on this description, the proposed hypothesis is as follows:

H1 Crude Oil Prices have a positive effect on Bitcoin Prices.

Rehman and Kang (2021) conducted a study whose results found that the bivariate co-movement of Bitcoin with the Natural Gas market revealed by the wavelet coherence test shows that Bitcoin has an equal and significant movement with Natural Gas. The Natural Gas Market is believed to have the ability to influence the Bitcoin market because the Bitcoin mining process relies heavily on energy. Derbali et al. (2020) also found a solid and significant dynamic conditional correlation between Bitcoin and the energy commodity market, where one of the energy commodities is Natural Gas. Symitsi and Chalvatzis (2018) conducted a study with VAR-BEKKAGARCH to measure the volatility between Bitcoin and Natural Gas energy commodities and found a significant influence of the energy commodity stock market on the Bitcoin market. Short-term volatility extends from Natural Gas to Bitcoin, while Bitcoin has a long-term volatility impact on the energy index. Goodell and Goutte (2020) research investigates how bitcoin and commodity markets relate. They use daily frequency data on Bitcoin and a bunch of commodities, one of which is gas. The study found that Bitcoin significantly impacted many commodities with a heterogeneous and more decisive response to financial stress. Azza et al. (2021) found positive and significant results in the short-term relationship between Bitcoin, Natural Gas, and the S&P 500. Rehman and Apergis (2019) also researches how the cryptocurrency market affects commodity markets and vice versa. The findings in this study reveal that cryptocurrency is one of the factors that can help explain the return and volatility behavior of certain commodity futures and vice versa. The results of this study also conclude that cryptocurrency returns need to be used in predicting commodity market returns and volatility, and vice versa. Therefore, commodity investors can consider and analyze the prevailing movements in the cryptocurrency market in their information collection while making investment decisions, and vice versa. Ghorbel and Jeribi (2021) conducted a study using the ARCH and GARCH methods regarding the energy sector and financial assets during Covid-19 and found a shock transmission effect from gas to Bitcoin; gas prices significantly positively affected the volatility of cryptocurrencies.

In contrast, the estimation of the GARCH method indicates a significant positive relationship between gas and cryptocurrency, resulting in the volatility of cryptocurrencies not only depending on past volatility but also the past volatility of gas prices. Tang and Aruga (2022) also researched Natural Gas and Bitcoin; the results found a short-term positive correlation of fewer than six months between Natural Gas and Bitcoin. Based on this description, the proposed hypothesis is as follows:

H2 Natural Gas Prices have a positive effect on Bitcoin prices.

## 3 Methodology

### 3.1 Data sources and observations

In this study, the method used is quantitative with a function to find secondary data in the form of weekly data of the time series data. In our paper, we only focus on secondary data. This study did not use primary data; this is because many previous studies with similar research topics only used secondary data as data in their research Azza et al. (2021); Das et al. (2020); Goutte & Keddad (2021); Jalal et al. (2020); Jin et al. (2019); Ozturk (2020); Rehman & Apergis (2019); Rehman & Kang (2021); S M Rashed Jahangir, (2018); and Symitsi & Chalvatzis (2018). In this regard, Jin et al. (2019) researched secondary data from bitcoin, gold, and oil variables. Bitcoin was earned from https://coinmarketcap.com/, gold was obtained from the Federal Reserve Bank of St. Louis, and oil was obtained from the Energy Information Administration (EIA). Das et al. (2020) researched bitcoin and crude oil using secondary data from Bloomberg. Goutte and Keddad (2021) also conducts research using bitcoin and commodity variables. The secondary data used is taken from Coinbase and Datastream. Jalal et al. (2020), who conducted a study on the role of commodity prices and the recognition of tax purposes on bitcoin prices, also used secondary data as the data used in their research. The secondary data are daily commodity prices taken from investing.com and bitcoin prices from coindesk.com. Rehman and Apergis (2019) conducted a study using secondary data from the Thomson Reuters Data Stream. Rehman and Kang (2021) also researches bitcoin and commodities by sourcing data from blockchain.com and the Thomson Reuters Data Stream. Symitsi and Chalvatzis (2018) conducted research by retrieving bitcoin and commodity secondary data from Datastream. Ozturk (2020) uses daily data from January 2, 2017, to 32 December 2019; all variable data is taken from Investing.com. Azza et al. (2021), taking the start date on 01/09/2019-30/04/2020, is daily data taken from yahoo finance and ourworldindata.org sources. S M Rashed Jahangir (2018) take research data sources from Indexmundi.

This period is determined based on research needs, namely historical data for the last 1.5 years. The variable used in this study is the price of Bitcoin as the dependent variable and the price of crude oil and natural gas prices as independent variables. We use weekly data, and all data is taken from finance.yahoo.com. First, we download the available data on the website, then move it into one excel sheet to make data processing easier. The filter function is also used to sort data from January 1, 2020, to July 31, 2021. After the data is downloaded and compiled in one excel sheet, the data is entered into the programs used, namely Stata 16 and SPSS. The data entered in the processing program will be converted into log values (log_variable), then descriptive statistical tests, classical assumption tests, and multiple linear regression can be done. Table 1 below shows the period and number of data observations.

**Table 1:** Period and number of data observations.

Variable	Period	Observation
Bitcoin Price	January 1, 2020–July 31, 2021	79
Crude Oil Price	January 1, 2020–July 31, 2021	79
Natural Gas Prices	January 1, 2020–July 31, 2021	79

All the variables above are raw data from the closing price (close price), and the number of observations for all variables is 79.

### 3.2 Sample collection method

The following techniques were employed to gather sample data for this study:

a Literature review, in which researchers looked up relevant theories and information about the subjects under discussion in earlier journals.
bA method of documentation whereby researchers gather information on the price of Bitcoin, Crude Oil, and Natural Gas for the 2020–2021 timeframe on Yahoo Finance from the official website (finance.yahoo.com).

### 3.3 Data analysis method

Stata 16 and SPSS were used in this work to carry out data analysis methodologies. Several tests, including multiple linear regression analysis, are run in this procedure, and the results are then validated using classical assumption tests including normality test, heteroscedasticity test, and multicollinearity test. Additionally, two types of hypothesis testing, known as simultaneous test (f test) and partial test (t-test), were used in this work. In this study, analysis was done to ascertain the impact of global commodity prices, particularly those for crude oil and natural gas, on the pricing of cryptocurrencies, particularly Bitcoin.

#### 3.3.1 Descriptive statistics

Researchers use descriptive statistics to present information and the characteristics of the variables used in this study. Descriptive statistics describe the variables used, which consist of the mean/mean, standard deviation, and maximum and minimum values.

#### 3.3.2 Multiple linear regression analysis

In this study, the data analysis technique used is multiple linear regression with the simple least squares method OLS (Ordinary Least Square) to determine the appropriate data model. OLS estimation is used so that the data becomes the best linear unbiased estimator (BLUE). Linear regression is one of the fundamental models in statistics that is used to determine the relationship between the independent variable and the dependent variable. The extension of this model is multiple linear regression. It shows the relationship between one dependent and several independent variables (Karafiath, 2009). The multiple regression model used in this study is as follows:

BTC = α + β1COP + β2NGP + ℇ

Description:

BTC = Bitcoin

α = Constant

β1, β2 = Multiple regression coefficients

COP = Crude Oil Price

NGP = Natural Gas Price

ℇ = Error term or error rate in research.

#### 3.3.3 Classical assumption test

The classical assumption test is one of the statistical requirements that must be carried out if a linear regression method is used. A classical assumption test is an assumption used to see if there are deviations in the regression model being tested. This study tested the classical assumptions related to normality, heteroscedasticity, and multicollinearity to avoid the problem of classical assumptions.

##### 3.3.3.1 Normality test

A normality test is a test carried out to assess the distribution of data in a group of data or variables and whether the distribution is normally distributed (Fahmeyzan et al., 2018). The normality test helps determine the data collected or taken from an average population. The normality test in this study was carried out using the Jarque-Bera statistical method. Jarque Bera is a normality test that measures whether the skewness and kurtosis of the sample are under the normal distribution. Jarque Bera is often used in the Normality Test on Residual variables resulting from the Linear Regression Test because of its excellent ability to detect normality in residuals.

##### 3.3.3.2 Heteroscedasticity test

Heteroscedasticity is the variance of one error with another that is different or not constant. We performed a heteroscedasticity test using the Glejser test method in this study. The Glejser test is carried out by regressing the independent variable with the residual. If the Glejser test results are significant, then heteroscedasticity occurs. Meanwhile, if the test results are insignificant, there is no heteroscedasticity. Significant and not significant can be determined through the significance value between the independent and residual variables. If the significance value is more than 0.05, then there is no heteroscedasticity problem in the regression model used in this study.

##### 3.3.3.3 Multicollinearity test

A normality test was conducted to determine the relationship between the independent variables. Multicollinearity is if two or more independent variables (independent) in regression are correlated (Mendenhall, 1996). Multicollinearity becomes a problem in regression analysis, especially in standard linear regression (OLS) (Dewi, 2010). The existence of high multicollinearity makes it impossible to see the effect of the independent variables on the response variables separately (Gujarati, 2010). The approach used to determine the presence or absence of multicollinearity in this study is the Variance Inflation Factor (VIF) test. The analysis is as follows:

1. If the tolerance value is >0.1 and VIF <10, then there is no multicollinearity in the regression model in this study.
2. If the tolerance value is <0.1 and VIF >10, there is a multicollinearity problem in the regression model in this study.

#### 3.3.4 Coefficient of determination

The coefficient of determination is carried out to measure how much independent influence is simultaneously on the dependent variable by looking at the adjusted R-Squared value. The value in the coefficient of determination is between 0 (zero) and 1 (one). The more the coefficient of determination value approaches one and away from zero, the more the independent variable can explain all the information needed to explain the dependent variable (Ghozali, 2016).

#### 3.3.5 Hypothesis test

##### 3.3.5.1 Simultaneous test (F test)

To determine if the independent variables had an impact on the dependent variable simultaneously, a simultaneous test (F test) was conducted (Prasetio, 2011). The alternative hypothesis (Ha) is accepted if F count > F table or probability significant value (Sig 0.05), according to the test procedures and criteria utilized in this study. The alternative hypothesis (Ha) is rejected if F count F table or probability significant value (Sig 0.05). If Ha is accepted, it signifies that the independent variable and the dependent variable are both significantly impacted at the same time. However, if Ha is rejected, it indicates that the independent variable has little effect on the dependent variable.

##### 3.3.5.2 Partial test (T-test)

The price of Crude Oil (X1) and the price of Natural Gas (X2) in this study are the independent variables. A partial test (t-test) is used to determine whether each independent variable has a positive and significant effect on the dependent variable, namely the price of Bitcoin (Y). The partial test is frequently employed to determine how much of the variance in the dependent variable can be accounted for by the independent variable. The partial test criteria employed in this study are if t count >0.05, which indicates that the independent variable has no effect on the dependent variable, then Ha (an alternative hypothesis) is rejected. The independent variable influences the dependent variable, according to the alternative hypothesis, Ha, which is accepted if the t count is less than 0.05.

### 3.4 Operationalization of variables

Variable Operationalization is needed to explain the variables used in research on the indicators that make it up so they can be measured. In this study, there are two variables: the independent variable, namely the price of Crude Oil and the price of Natural Gas, and the dependent variable, namely the price of Bitcoin. The operationalization of variables in this study can be seen in Table 2 below:

**Table 2:** Variable operationalization table.

No	Variable	Data Category	Operational definition
**Dependent Variable**			
1	BTC_USD (Y)	Bitcoin Price	Bitcoin price converted in United States Dollar (USD)
**Independent Variable**			
1	Crude Oil Price	Macroeconomic Indicators	Crude Oil Price, measured from the spot price of the world oil market, in dollars per barrel. (USD)
2	Natural Gas Prices	Macroeconomic Indicators	Natural Gas Prices are obtained from the New York Mercantile Exchange commodity futures exchange.

## 4 Results and discussion

### 4.1 Overview of sample data

In this study, the method used is a quantitative method with secondary data as the data type used. The research was conducted on cryptocurrencies, especially Bitcoin and Crude Oil and Natural Gas, from January 2020–June 2021. In this study, we started data processing with Descriptive Statistics analysis and then continued on the Classic Assumption Test. The classical Assumption Test used in this research is Normality Test, Multicollinearity Test, and Heteroscedasticity Test. Then the coefficient of determination was also carried out, and, in this study, Partial Hypothesis Testing and Simultaneous Hypothesis Testing were used.

### 4.2 Descriptive statistics

In this study, the variable used is the price of Bitcoin as the dependent variable, then the price of Crude Oil and the price of Natural Gas as the independent variables. Table 3 above shows the results of statistical calculations processed using the Stata 16 program, and Table 4 shows the results of statistical calculations processed using the SPSS 23 program. Tables 3 and 4 above show the data used in this study (Obs), totaling 79 data. This amount is data from Bitcoin prices, Crude Oil prices, and Natural Gas prices for January 2020–June 2021. It can be seen that the results from the descriptive statistics table produced by the two programs are not much different and almost the same. The Bitcoin (BTC) price variable has a maximum value of 11,05885, a minimum value of 8.561331, the average value (mean) is 9.755214, and the standard deviation value is 0.747982. The data processing results with the Stata 16 program show that the standard deviation value is smaller than the mean value. This means that the spread of Bitcoin price data is evenly distributed, and there is no significant difference between the data. The crude oil price variable has a maximum value of 4.319619, a minimum value of 2.303585, an average (mean) of 3.799228, and a standard deviation of .3761343. The data processing results with the Stata 16 program show that the standard deviation value is smaller than the mean value. This shows the same as Bitcoin prices, where the spread of Crude Oil price data is evenly distributed, and there is no significant difference between the data. The natural gas price variable has a maximum value of 1.308333, a minimum value of 0. 4762342, the mean (mean) is .8507919, and the standard deviation is .2283004. The data processing results with Stata 16 and SPSS 23 programs show that the standard deviation value is smaller than the mean value. This means that the distribution of Natural Gas price data is evenly distributed, and there is no significant difference between the data.

**Table 3:** Descriptive statistics (stata 16).

Variable	Obs	Mean	Std. Dev	Min	Max
log_BTC	79	9.755214	.747982	8.561331	11.05885
log_CO	79	3.799228	.3761343	2.303585	4.319619
log_NG	79	.8507919	.2283004	.4762342	1.308333

**Table 4:** Descriptive statistics (SPSS 23).

Descriptive Statistics					
	N	Minimum	Maximum	mean	Std. Deviation
ln_BTC	79	8.56	11.06	9.7552	.74798
ln_CO	79	2.30	4.32	3.7992	.37613
ln_NG	79	.48	1.31	.8508	.22830
Valid N (listwise)	79				

### 4.3 Normality test

**Figure 1:**
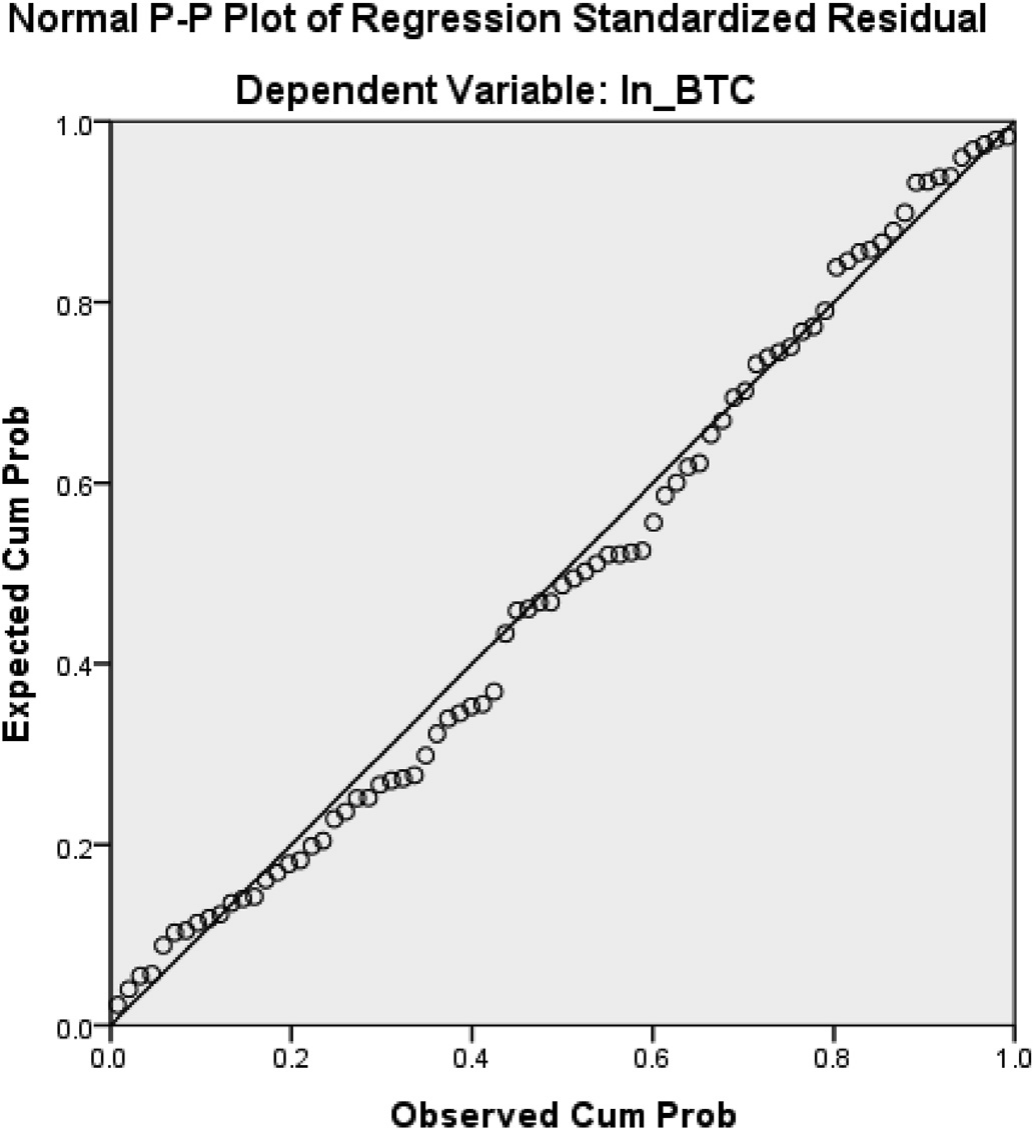
Normal PP plot.

A normality test is performed to determine whether the data collected is normally distributed or taken from an average population. Based on Table 5, which is the result of processing using the Stata 16 program, it can be seen that the Bitcoin value on the probability is 0.0000. As well as, the value of Natural Gas on the probability is 0.0000. The value of Crude Oil on the probability is 0.000. The three variables show numbers that are smaller than 0.05. This indicates that the Bitcoin, Crude Oil, and Natural Gas data are not normally distributed. However, based on Table 6 and Figure 1 processed using SPSS 23, it can be seen that the results show that the data used are typically distributed. It can be seen from Table 6 that the significant value generated by Asymp. Sig. (2-tailed) is greater than the significance level of 0.05. This means that according to the decision-making criteria in the Kolmogorov-Smirnov test, it can be concluded that the data is normally distributed so that it meets the assumption of normality. Figure 1 also shows that the data used is normally distributed. This is because the data in the image shows a distribution close to the line so that it can be interpreted that the data used is normally distributed (Ghasemi and Zahediasl, 2012).

**Table 5:** Normality test (stata 16).

Skewness/Kurtosis tests for Normality					
				------ joint ------	
Variable |	Obs	Pr (Skewness)	Pr (Kurtosis)	adj chi2(2)	Prob>chi2
**-------------+--------------------------------**					
log_BTC |	79	0.1078	0.0000	39.75	0.0000
log_CO |	79	0.0000	0.0007	24.39	0.0000
log_NG |	79	0.6383	0.0000	25.64	0.0000

**Table 6:** Normality test (SPSS 23).

One-Sample Kolmogorov-Smirnov Test		
		Unstandardized Residual
N		79
Normal Parameters, b	Mean	.0000000
	Std. Deviation	.42572645
Most Extreme Differences	Absolute	.070
	Positive	.070
	Negative	−.049
Test Statistics		.070
asymp. Sig. (2-tailed)		.200c,d

a. Test distribution is Normal.

b. Calculated from data.

c. Lilliefors Significance Correction.

d. This is a lower bound of the true significance.

### 4.4 Heteroscedasticity test

The heteroscedasticity test detects whether there is an inequality of variance from the residuals of one observation with another. If there is an inequality in the variance, it can be concluded that there is a heteroscedasticity problem. A variable is considered free from heteroscedasticity if the value of prob > chi 2 is greater than 0.05. The results of Table 7 show that the value of prob > chi 2 is 0.0791, meaning there are no symptoms of heteroscedasticity in the data used with the Stata 16 program. In Table 8, it can be seen from the sig value that if the value is smaller than 0.05, then heteroscedasticity occurs. The sig value obtained by Crude Oil is 0.725 while for Natural Gas is 0.10. These results show differences in the results of the Stata 16 and SPSS 23 programs.

**Table 7:** Heteroscedasticity test (stata 16).

Breusch-Pagan/Cook-Weisberg test for heteroskedasticity
Ho: Constant variance
Variables: fitted values of log_BTC
chi2(1) = 3.08
Prob > chi2 = 0.0791

**Table 8:** Heteroscedasticity test (SPSS 23).

Coefficients						
Model		Unstandardized Coefficients		Standardized Coefficients	t	Sig.
		B	Std. Error	Beta		
1	(Constant)	.133	.282		.471	.639
	ln_CO	−.032	.090	−.049	−.353	.725
	ln_NG	.393	.149	.368	2,642	.010

a. Dependent Variable: Abs_RES.

### 4.5 Multicollinearity test

The multicollinearity test for this study was conducted by examining the VIF (variance inflation factor) values in Tables 9 and 10 above. The VIF column of Tables 9 and 10 above shows the VIF value of each independent variable. Based on Tables 9 and 10, all the independent variables used in this study, namely the price of Crude Oil and Natural Gas in the regression model above, have a VIF value of 1.66, which is below 10. This means that the regression model formed and used in this study does not have a multicollinearity problem. The absence of this multicollinearity problem means that the regression model used is good because there is no correlation between the independent variables. The tolerance value can also show whether there is multicollinearity in data. If the tolerance value is greater than 0.1, there is no multicollinearity. It can be seen in Table 10 that the tolerance value is 0.601, which indicates that there is no multicollinearity.

**Table 9:** Multicollinearity test (stata 16).

Variable	VIF	1/VIF
log_CO	1.66	0.600846
log_NG	1.66	0.600846
Mean VIF	1.66

**Table 10:** Multicollinearity test (SPSS 23).

Coefficients								
Model		Unstandardized Coefficients		Standardized Coefficients	t	Sig.	Collinearity Statistics	
		B	Std. Error	Beta			Tolerance	VIF
1	(Constant)	5.415	.523		10.351	.000		
	ln_CO	.755	.167	.380	4.509	.000	.601	1,664
	ln_NG	1,729	.276	.528	6.266	.000	.601	1,664

a. Dependent Variable: ln_BTC.

### 4.6 Multiple linear regression

The method used in this research is multiple linear regression. This multiple linear regression is used to determine whether there is an effect of the independent variable on the dependent variable. The resulting coefficients are not much different based on Table 11 and Table 12 above. So, the equation of the multiple linear regression model obtained is as follows:

**Table 11:** Multiple linear regression (stata 16).

Source	SS	Df	MS	Number of Obs = 79		
				F (2, 76) = 79.30		
Model	29.5022585	2	14.7511292	Prob > F = 0.0000		
Residual	14.1369515	76	.18601252	R-Squared = 0.6760		
				Adj R-squared = 0.6675		
Total	43.63921	78	.559477051	Root MSE = .43129		

log_BTC	Coef.	Std. Err.	t	P > |t|	[95% Conf. Interval]	

log_CO	.7552972	.167494	4.51	0.000	.421704	1.08889
log_NG	1.729056	.2759532	6.27	0.000	1.179448	2.278665
_cons	5.4146	.5231016	10.35	0.000	4.372753	6.456447

**Table 12:** Multiple linear regression (SPSS 23).

Model		Unstandardized Coefficients		Standardized Coefficients	t	Sig.
		B	Std. Error	Beta		
1	(Constant)	5.415	.523		10.351	.000
	ln_CO	.755	.167	.380	4.509	.000
	ln_NG	1,729	.276	.528	6.266	.000

Y = Bitcoin = 5.4146 + 0.7552972 Crude Oil +1.729056 Natural Gas.

The equation of the multiple linear regression model above shows a constant value of 5.4146. As for the independent variable Crude Oil is equal to 0.7552972, and for the independent variable, Natural Gas is equal to 1.729056. From the equation above, we can understand that if the independent variable Crude Oil and Natural Gas does not carry out operational activities or is worth 0 in the research period, namely January 1, 2020–June 30, 2021, then the value of Bitcoin is 5.4146.

The crude Oil variable has a positive coefficient value of 0.7552972. This means that every 1% increase in Crude Oil will increase the value of Bitcoin by 0.0075 with other variables held constant. On the other hand, the Natural Gas variable has a positive coefficient value of 1.729056. This means that every 1% increase in Crude Oil will increase the value of Bitcoin by 0.017 with other variables held constant.

### 4.7 Coefficient of determination

Based on Table 13 above, it can be seen that the value of the correlation coefficient (R) is 0.676. This means that the relationship between the independent variable and the dependent variable is 67.6%, indicating that the relationship between the independent variable and the dependent variable is quite strong. In contrast, the value of Adjusted R Square is 0.668. This value indicates that the independent variables in this study, namely Crude Oil and Natural Gas, can explain 66.8% of the variation of the dependent variable, which in this study is bitcoin. On the other hand, there is still a 33.2% variation in the bitcoin variable, which is explained by other variables outside this study.

**Table 13:** Coefficient of determination (SPSS 23).

Model Summary				
Model	R	R Square	Adjusted R Square	Std. Error of the Estimate
1	.822a	.676	.668	.43129

a. Predictors: (Constant), ln_NG, ln_CO.

b. Dependent Variable: ln_BTC.

### 4.8 Hypothesis test

#### 4.8.1 Partial hypothesis test (t-test)

Partial hypothesis testing aims to test whether the independent variables in this study, Crude Oil and Natural Gas, partially affect the dependent variable, namely Bitcoin. The test criteria in the partial hypothesis test are if the probability value < 0.05, then the hypothesis is accepted. Based on Table 11 and Table 12, the effect of Crude Oil on Bitcoin has a probability value of 0.00, which is smaller than 0.05. Therefore, we can conclude that Crude Oil has a positive and significant effect on Bitcoin, so H1 is accepted. On the other hand, the effect of Natural Gas on Bitcoin also has a probability value of 0.00, which is smaller than 0.05. Therefore, we can conclude that Natural Gas has a positive and significant effect on Bitcoin, so H2 is accepted.

#### 4.8.2 Simultaneous Hypothesis Testing (F test)

The F test was conducted to simultaneously determine the independent variables' effect by comparing the F table. Based on Table 11, which was processed using Stata 16, and Table 14, which was processed using SPSS 23, the regression results of the data obtained were not much different. From the results obtained, the F statistic value is 79.30, with a probability value of 0.0000 > 0.05. Because the probability is much smaller than 0.05, it can be concluded that there is a simultaneous effect of Crude Oil and Natural Gas on Bitcoin of 66.75% seen from the Adjusted R Squared. The calculated F table is 3.11, so the calculated F is greater than the F table, so the H1 and H2 hypotheses are accepted.

**Table 14:** Simultaneous test (SPSS 23).

ANOVA						
Model		Sum of Squares	Df	Mean Square	F	Sig.
1	Regression	29,502	2	14,751	79.302	.000b
	Residual	14.137	76	.186		
	Total	43,639	78			

a. Dependent Variable: ln_BTC

b. Predictors: (Constant), ln_NG, ln_CO

### 4.9 Hypothesis testing and discussion

#### 4.9.1 Effect of crude oil price on bitcoin price

The probability value of the Crude Oil price variable is 0.00 according to the output of the t-test that has been performed; where the probability value is less than 0.05, H1 is accepted. The output concludes that the Crude Oil price variable positively affects Bitcoin price movements. This means that the volatility of Crude Oil will affect the price movement of Bitcoin itself. For instance, the volatility of crude oil might influence the volatility of bitcoin when the price of crude oil rises or falls.

As we know, during this Covid-19 pandemic, Bitcoin experienced significant price fluctuations in 2021. One reason for that is that bitcoin transactions occur every day, which allows prices to rise or fall drastically. Also, in the cryptocurrency market, there is no cooling-off period like in equity, such as bonds or stocks, which is the period between issuing a prospectus and selling new stock or bond offerings. However, fluctuations that have a negative connotation can have an impact in the opposite direction. 60% of Bitcoin's profit this year, making Bitcoin one of the best-performing assets in 2021 (Hajric and Greifeld, 2021). Based on the results of our research which shows the effect of crude oil prices on bitcoin prices in the long term, it can be said and assumed that there is a role for crude oil prices to fluctuate in Bitcoin prices during the year 2021.

Results in this research are supported by the results of research conducted by Kaabia et al. (2020) found that the Crude Oil variable has a robust correlation (greater than 0.8). Moreover, the empirical results state that a significant positive relationship exists between Bitcoin and Crude Oil prices. The results confirm that Crude Oil prices remain sensitive to Bitcoin prices; therefore, the entire energy industry will be affected. Okorie & Lin (2020) Also conducted research using the VAR MGARCH GJR–BEKK and Wald test methods to measure the volatility relationship between Crude Oil and Bitcoin. And it was found that there is a bidirectional relationship and unidirectional volatility from the Crude Oil market to the Cryptocurrency market. The multivariate portmanteau test also shows that cryptocurrency returns with Crude Oil have serial cross-correlation, even as a lag order of 10. It was also found that there is significant volatility persistence between the Crude Oil market and the Cryptocurrency market. The Wald test statistics also show that the Crude Oil market and the Cryptocurrency market are connected by volatility; therefore, there is an abundance of directional volatility (unidirectional and bidirectional) between the Crude Oil market and the Cryptocurrency market.

Ozturk (2020) also conducted a similar study on the relationship between Bitcoin, Crude Oil, and Gold and found a higher volatility relationship than the return relationship on the three variables. The relationship in volatility mainly occurs at medium frequencies, although the relationship in returns is mostly at high frequencies. The overall relationship hit a high of around 70% in November 2017 when price movements started to pick up, and volatility was high. Compared to the volatility relationship, the relationship between returns is not as high as volatility and is around 5% and 35%, and the highest peak is in the October and December 2019 periods. So, the percentage of the relationship between volatility is much higher, almost double the correlation compared to returns. The results in this study are also in accordance with research conducted by Jin et al. (2019), who conducted a study that aims to identify which is more informative in determining price fluctuations using bitcoin, gold, and crude oil.

Furthermore, it was found that the spillovers from the gold and crude oil markets to the Bitcoin market were much more substantial than the other spillovers, indicating a relationship between Bitcoin and crude oil. The results of our study found that the price of crude oil has a relationship with the price of bitcoin, so that it can support the study conducted by Ciaian et al. (2016) stated that the price of Crude Oil is considered a determinant of Bitcoin volatility. Moreover, it can also support the results of studies conducted by Vassiliadis (2017), which prove that Bitcoin has a cross-correlation with the price of Crude Oil and Gold. Van Wijk (2013) conducted a study examining the value of bitcoin using financial data, one of which was oil price. The oil prices used in this study are the Brent oil price, the West Texas Intermediate oil price, and the UBS Bloomberg Constant Maturity Commodity Index of Oil. The results of this study state that the price of WTI oil significantly influences the value of Bitcoin in the long term; this proves that our research has the same results as previous researchers have done.

#### 4.9.2 Effect of natural gas prices on bitcoin prices

H2 is approved since the probability value of the Natural Gas price variable is 0.01, which is lower than the threshold of 0.05 based on the output of the t-test explained in the previous sub-chapter. The output concludes that the Natural Gas price variable has a positive effect on the Bitcoin variable. These results show that natural gas price movements affect Bitcoin price movements. This means that the volatility of Natural Gas will affect the price movement of Bitcoin; for example, when the price of Natural Gas goes up or down, then the volatility of Natural Gas can affect the volatility of Bitcoin.

Bitcoin was the most traded asset in 2021. However, the price of Bitcoin itself will also fluctuate during 2021. The lowest bitcoin price was recorded at $28,803.59 on January 1, 2021, while the highest was $68,789.63 on November 10, 2021. The difference between the lowest and highest prices for bitcoin is 138.8% (Dey, 2021). This is a huge number and reflects just how significant the fluctuations in the price of bitcoin are and the uncertainty of bitcoin in the future. Our research results found that there is a positive influence between commodity prices, especially natural gas, on bitcoin prices. It can be assumed that there is a role for commodities, especially natural gas, in bitcoin price fluctuations during 2021.

Results in this study support the results of research conducted by Rehman and Kang (2021), which states that the price of Natural Gas influences the price of Bitcoin. His research stated that Natural Gas influences Bitcoin because the Bitcoin mining process depends on the energy itself. The results of this study also support the results of research conducted by Derbali et al. (2020). They found a strong and significant correlation between Bitcoin and energy commodity markets if monetary policy is included in the variance. The study also states that the behavior of each commodity concerning Bitcoin fluctuations suggests that commodities cannot be seen as a homogeneous asset class. The results in this study on the regression value are the same as the research conducted by Azza et al. (2021), which found a positive relationship in the short term between Bitcoin, Natural Gas, and the S&P 500. This means that it also supports research conducted by Tang and Aruga (2022), who researched Natural Gas and Bitcoin. The results found a short-term positive correlation for less than six months between Natural Gas and Bitcoin; although our research has a more extended period of 1.5 years, the results obtained are the same, namely the positive discovery correlation between Natural Gas and Bitcoin. We used a different method, namely multiple linear regression, but the results found were the same as with the study conducted by Ghorbel and Jeribi (2021), who conducted a study using the ARCH and GARCH methods regarding the energy sector and financial assets during Covid-19 and found that there was a shock transmission effect from gas to Bitcoin, gas prices significantly positively affected the volatility of cryptocurrencies.

## 5 Conclusion and suggestions

This paper investigates the relationship between Bitcoin and two commodities, namely Crude Oil and Natural Gas, for January 1, 2020–July 31, 2021. The data source we use in this study is finance.yahoo.com. Regarding methodology, we used multiple linear regression to analyze each variable in the Stata 16 and SPSS 23 programs. Classical assumption tests were also carried out, including normality, multicollinearity, heteroskedasticity, and coefficient of determination. Based on some of the analyzes we did; it was found that there is a positive relationship between Crude Oil on Bitcoin and Natural Gas on Bitcoin. It is undeniable that the price of Crude Oil and Natural Gas has a significant influence on the price movement of Bitcoin itself. This is because, in mining which Bitcoin miners often do, energy is needed that can help the Bitcoin mining process. Therefore, when the Bitcoin mining process increases, the energy consumption required will also increase, and commodity prices will also increase.

This study finds a relationship between Bitcoin and two energy commodities, namely Crude Oil and Natural Gas, which is expected to provide insight to the government in making regulations related to Cryptocurrencies. Because lately, it has been much talk. Although assets from cryptocurrency are in great demand in Indonesia, transactions in Indonesia are still relatively small, which is only 1% of global volume transactions. With the results of this study, it is hoped that the government can also consider that cryptocurrency can be used as a means of payment because cryptocurrency has a positive relationship with the commodity, especially crude oil and natural gas, and how the cryptocurrencies are reshaping the world's financial industry, especially the biggest cryptocurrency, Bitcoin. If the regulations for Bitcoin are clear and allow for payment instruments, then it is possible that the government can make Bitcoin a taxes target in order to increase state revenue and development for the state. The results in this paper are also expected to help and become a source of information for investors investing in Cryptocurrencies, especially in reading the direction of the price movement of Bitcoin and two commodities, namely Crude Oil and Natural Gas, in turbulent and uncertain periods. However, it must be noted that other things can affect these three variables, such as news, the state of a country's economy, and other things. The energy-consuming process of Bitcoin mining and the uncertainty and high risk of the Cryptocurrency market, exacerbated by the Covid-19 pandemic, has attracted the interest of researchers in researching the Cryptocurrency market and its relationship to commodity markets. Therefore, investors can use the commodity market as a reference in determining their decision to invest in the Cryptocurrency market.

The novelty of this research is that we use two data processing programs, namely Stata and SPSS, to strengthen the analysis results. The time-taking range differed from previous studies; we used 18 months or 1.5 years. The use of a long period is expected to provide a clearer picture of the relationship of two energy commodities, namely Crude Oil and Natural Gas, to Bitcoin. Then the last is that we use multiple linear regression methods that are different from previous studies; it is hoped that using different methods can find more precise analytical results.

Based on the results of the hypothesis testing, there will be analysis, discussion, and a list of some of its limitations. Readers may be offered recommendations; it is intended that this research will add perspective and information regarding how changes in the price of crude oil and natural gas affect changes in the price of bitcoin. Additionally, it is anticipated that input for future researchers would be able to utilize various research periods. In order to acquire alternative results, it can also use other independent factors from this research as well as additional dependent variables as research samples. It is also recommended for further researchers to use other methods such as the ordinary least square, fixed effect model, random effect model, dynamic panel data, ARCH/GARCH, structural equation model, and logistic regression. By using different methods, it is expected to find different results.

## Declarations

### Author contribution statement

MEIRYANI Meiryani: conceived and designed the experiments; performed the experiments; analyzed and interpreted the data; contributed reagents, materials, analysis tools or data; wrote the paper.

Caineth Delvin: conceived and designed the experiments; performed the experiments; wrote the paper.

Jason Emanuel: conceived and designed the experiments; performed the experiments; contributed reagents, materials, analysis tools or data; wrote the paper.

ASL Lindawati: contributed reagents, materials, analysis tools or data.

Mochammad Fahlevi: analyzed and interpreted the data; wrote the paper.

Mohammed Aljuaid: conceived and designed the experiments.

Fakhrul Hasan: performed the experiments.

### Funding statement

This work was supported by King Saud University through the Researcher Supporting Project (RSP2022R481), King Saud University, Riyadh, Saudi Arabia.

### Data availability statement

Data included in article/supp. material/referenced in article.

### Declaration of interest’s statement

The authors declare no conflict of interest.

### Additional information

No additional information is available for this paper.
